# The Effects of Different Preoperative Biliary Drainage Methods on Complications Following Pancreaticoduodenectomy

**DOI:** 10.1097/MD.0000000000000723

**Published:** 2015-04-10

**Authors:** Xin Huang, Bin Liang, Xiang-Qian Zhao, Fu-Bo Zhang, Xi-Tao Wang, Jia-Hong Dong

**Affiliations:** From the Department and Institute of Hepatobiliary Surgery (XH, BL, XQZ, FBZ, XTW, JHD), Chinese PLA General Hospital, Beijing, China; and School of Medicine (XH, FBZ, XTW), Nankai University, Tianjin, China.

## Abstract

The objective of this study was to investigate the effects of different preoperative biliary drainage (PBD) methods on complications in jaundiced patients following pancreaticoduodenectomy. We retrospectively analyzed 270 extrahepatic bile duct cancer patients who underwent pancreaticoduodenectomy. A total of 170 patients without PBD treatment were defined as the non-PBD group. According to different PBD methods, 45, 18, and 37 patients were classified into the percutaneous transhepatic biliary drainage (PTBD), endoscopic nasobiliary drainage (ENBD), and endoscopic retrograde biliary stent (ERBS) groups, respectively. Clinical characteristics and complications were compared among the 4 groups.

Preoperative cholangitis occurred in 14 (8.2%) and 8 (21.6%) patients in the non-PBD and ERBS group, respectively (*P* *=* 0.04). Compared with the non-PBD group, delayed gastric emptying (DGE) and wound infection occurred significantly more often in the ERBS group. The incidence of severe complications was significantly lower in the PTBD group than the non-PBD group (*P* *=* 0.03). Postoperative hospital stay and complication rates were significantly higher in the ERBS group than the PTBD group. There were no significant differences in complications between ENBD and other groups.

In conclusion, PTBD can improve surgical outcomes by reducing severe complication rate in jaundiced patients following pancreaticoduodenectomy. ERBS increased the rates of DGE and wound infection due to high incidence of cholangitis before operative intervention and should be avoided. ENBD carried no special effect on complications and needs further analysis.

## INTRODUCTION

The discussion on the use of preoperative biliary drainage (PBD) in jaundiced patients who will be candidates for a resection approach with curative intent has lasted for years. The theories which support PBD as a routine procedure consider that obstructive jaundice is associated with hepatic dysfunction, disturbances in coagulation, and the development of bacterial translocation and cholangitis. PBD can effectively decrease serum bilirubin levels and reverse those pathological procedures.^1,2^ Several clinical studies revealed that PBD could improve the resection rate of patient and decrease morbidity and mortality after surgery.^2–4^ On the contrary, some studies reported that PBD carried no benefit in improving patient outcome but increasing rates of certain complications and should be routinely avoided in patients with potentially curative surgery.^5,6^ The efficiency of PBD is still controversial.

If PBD is indicated for jaundiced patients, the optimal drainage method is unclear. Nowadays, there are 3 commonly used methods for achieving PBD – percutaneous transhepatic biliary drainage (PTBD), endoscopic nasobiliary drainage (ENBD), and endoscopic retrograde biliary stent (ERBS). In various studies concerning clinical outcome of biliary drainage, different PBD methods were treated as a whole group, ignoring the effects of individual differences of these methods on perioperative complications.^7–9^ In fact, a prospective randomized trial revealed that preoperative biliary stent in patients with pancreatic head cancer increased the rate of overall complications and was best avoided.^10^ Another study reported that PTBD with bile re-infusion improved the resection rates and showed a good safety profile in patients with hilar cholangiocarcinoma.^11^ Combining the analysis methods will balance the advantages and disadvantages of different PBD methods, leading to variable conclusions. Therefore, the effect of biliary drainage on patient outcome should be analyzed in accordance with different PBD methods.

In this study, we retrospectively evaluated clinical characters and outcomes in extrahepatic bile duct cancer patients following pancreaticoduodenectomy to investigate the effects of different PBD methods on perioperative complications.

## PATIENTS AND METHODS

From January 2005 to June 2014, patients who underwent pancreaticoduodenectomy were selected from a prospectively maintained database at Chinese PLA General Hospital. To avoid skewed results by confounding variables, only patients with extrahepatic bile duct cancer, which was confirmed by the postoperative pathological diagnosis, were included. Details of 270 eligible patients including demographics, types of biliary drainage, biochemical laboratory tests, surgery, pathology, perioperative complications, and postoperative hospital stay were retrospectively collected. This study was performed according to the guidelines of the Medical Ethics Committee of Chinese PLA General Hospital.

The choice of performing biliary drainage and the suitable approach of PBD for each patient was decided by the surgeon. There are several indications for extrahepatic bile duct carcinoma patients performing PBD, which are as follows: patients with total bilirubin (TB) >256 umol/L, patients with poor physical conditions due to obstructive jaundice, and jaundiced patients with cholangitis. The selection of PBD methods depends on the surgeon's experience and patient's wish. The endoscopic biliary drainage methods (ENBD and ERBS) were performed using standard procedures with or without endoscopic sphincterotomy. A 10-Fr plastic stent was used in the ERBS group and a 7-Fr tube in the ENBD group. PTBD was achieved under ultrasound guidance with a 10-Fr tube. The end of PTBD catheter was usually placed into the hilar bile duct to avoid contact with the tumor. Once stent/tube occlusion occurred, it would be replaced with the same procedure. PBD duration was defined as the time interval between the first drainage procedure and pancreaticoduodenectomy. If no contraindications for resection were found, patients underwent either the standard Whipple's operation (PD) or the pylorus-preserving pancreaticoduodenectomy (PPPD). The pancreatico-enteric anastomosis was done by either end-to-side pancreaticojejunostomy or duct to mucosa technique. Routinely, 2 closed suction drains were placed in the pancreatic anastomosis area.

Among the 270 patients, 170 who underwent pancreaticoduodenectomy without PBD were defined as the non-PBD group. The remaining 100 patients were classified by different PBD methods, including the PTBD group (n = 45), ENBD group (n = 18), and ERBS group (n = 37). The procedures of PBD were successfully performed in patients in the drainage groups. Stent/tube occlusion occurred in 3 patients (2 ERBS and 1 PTBD group) and all of them were successfully replaced with the same procedure. None of the patients received preoperative chemotherapy or radiotherapy which was not recommended in our center. Tumor location was defined according to the position of main tumor: middle (between inferior hilar and superior pancreas) and distal (inside pancreas). Differences in clinical characteristics and perioperative complications among the 4 groups were first analyzed. Subsequently, pairwise comparisons of the groups were performed in parameters with possible differences to find out the specific differences between the 4 groups.

### Definition of Complications

Perioperative complications consisted of preoperative and postoperative complications. All complications were classified in accordance with Clavien–Dindo classification.^12^ Severe complications were defined as a condition that was grade III or more based on the Clavien–Dindo classification. Morbidity and mortality were defined as complications or death occurring either within 30 days from the operation or during the hospital stay. Preoperative complications included cholangitis, pancreatitis, hemorrhage, perforation, stent/tube occlusion, and catheter tract implantation. Cholangitis was defined by new diagnostic criteria of acute cholecystitis and cholangitis referred to as the Toyko Guidelines.^13^ Pancreatitis was considered to be present if the patient developed upper abdominal pain with a serum concentration of pancreatic enzymes 3 or more times the upper limit of normal.

Postoperative complications included pancreatic fistula (PF), delayed gastric emptying (DGE), postpancreatectomy hemorrhage (PPH), intra-abdominal infection, sepsis, wound infection, and bile leakage. Other general complications, such as pneumonia and renal dysfunction, were also evaluated. The diagnosis and grade of PF, PPH, and DGE were confirmed by standards from the International Study Group on Pancreatic Surgery.^14–16^ The grades of these complications were defined according to clinical conditions, comorbidities, treatments, and so on. Infectious complications were defined as any complication with evidence of associated localized or systemic infection indicated by fever and high white blood cell count, and confirmed by imaging techniques or positive culture. Bile leakage was determined when bilirubin level was 3 or more times higher than that of serum bilirubin level in the drain fluid.

### Statistical Analysis

Data were expressed as means ± SD or median (range). The statistical analyses of categorical data were performed using the χ^2^ test with Yates’ correction or Fisher exact test where appropriate. One-way analysis of variance with Dunnett's post hoc test and LSD test was performed for the comparison of numeric data. The Statistical Package for Social Sciences version 17.0 software (SPSS Inc., Chicago, IL) was used for all the statistical analyses. A 2-tailed *P* value <0.05 was considered to be statistically significant.

## RESULTS

### Comparisons of Clinical Characteristics

Clinical characteristics of patients are shown in Table 1 and pairwise comparisons between the 4 groups in parameters with possible differences are shown in Table 2. There were no significant differences in age, gender, operative procedure, operative time, intraoperative bleeding, blood transfusion, tumor size, and location among the 4 groups. Preoperative alanine aminotransferase (ALT) and TB levels were significantly higher in the non-PBD group than other groups, and no significant differences were observed between the drainage groups. However, PBD duration was significantly longer in the ERBS group than other 2 groups (PTBD vs ERBS: *P* *=* 0.04; ENBD vs ERBS: *P* *=* 0.03). It appeared that patients with ERBS treatment spent longer time waiting for operations. The longest postoperative hospital stay was also found in the ERBS group, with a significant difference among the 4 groups (*P* *=* 0.04). Compared with the ERBS group, postoperative hospital stay was significantly shorter in the non-PBD group (*P* *=* 0.02) and PTBD group (*P* *=* 0.02).

**TABLE 1:**
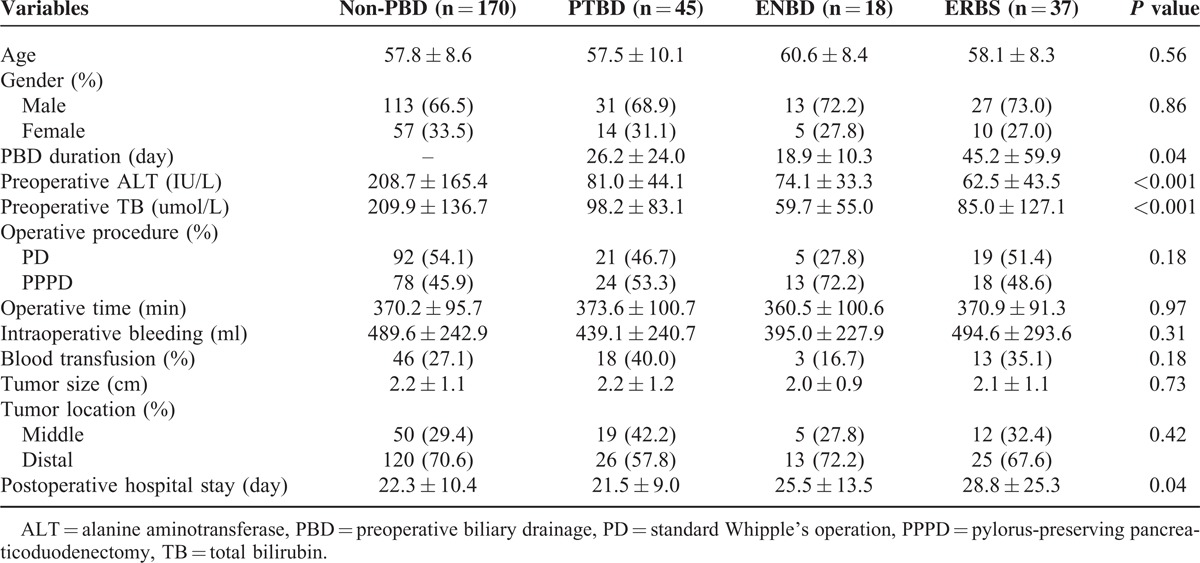
Comparisons of Clinical Characteristics Among the 4 Groups

**TABLE 2:**
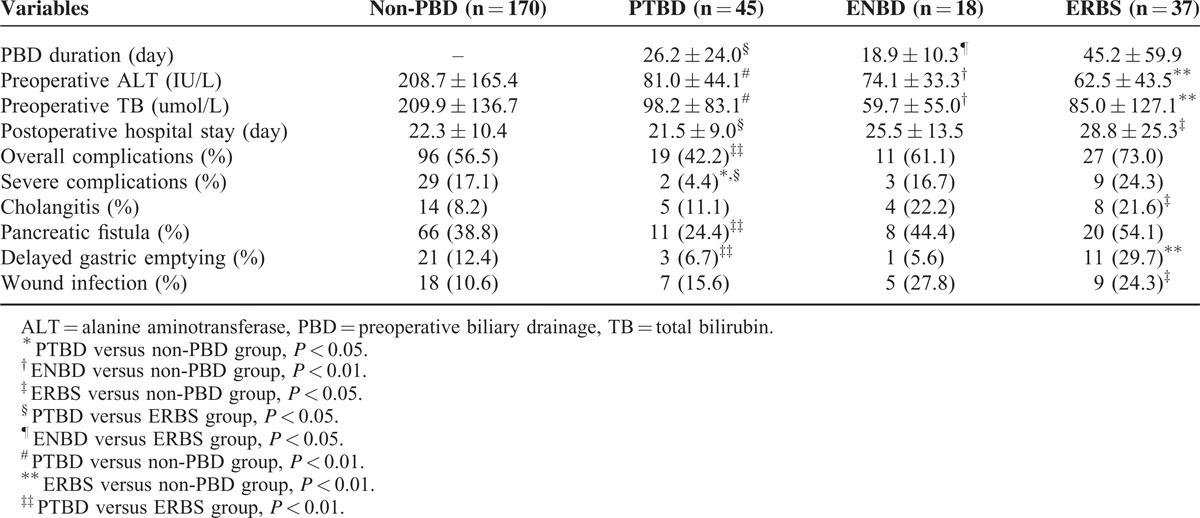
Pairwise Comparisons Between the 4 Groups in Parameters With Possible Differences

### Comparisons of Perioperative Complications

Perioperative complications of the 4 groups are summarized in Table 3. Preoperative complications including hemorrhage, perforation, and catheter tract implantation were not found in this study. Significant differences among the groups were detected in overall complications (*P* = 0.04) and DGE (*P* = 0.01). Besides overall complications and DGE, severe complications, cholangitis, PF, and wound infection were added to further analysis (Table 2). Severe complications developed in 29 (27.1%) patients in the non-PBD group and in 2 (4.4%) patients in the PTBD group, with a significant difference (*P* *=* 0.03). It revealed that PTBD could effectively reduce the incidence of severe complications following pancreaticoduodenectomy. Significant difference in preoperative cholangitis was only observed between the non-PBD group and ERBS group (*P* *=* 0.04). Meanwhile, DGE and wound infection occurred significantly higher in the ERBS group than the non-PBD group (DGE: 29.7% vs 12.4%, *P* *=* 0.008; wound infection: 24.3% vs 10.6%, *P* *=* 0.04). Compared with the PTBD group, the rates of overall and severe complications, PF, and DGE were significantly higher in the ERBS group. Interestingly, there were no significant differences in complications between the ENBD group and other groups.

**TABLE 3:**
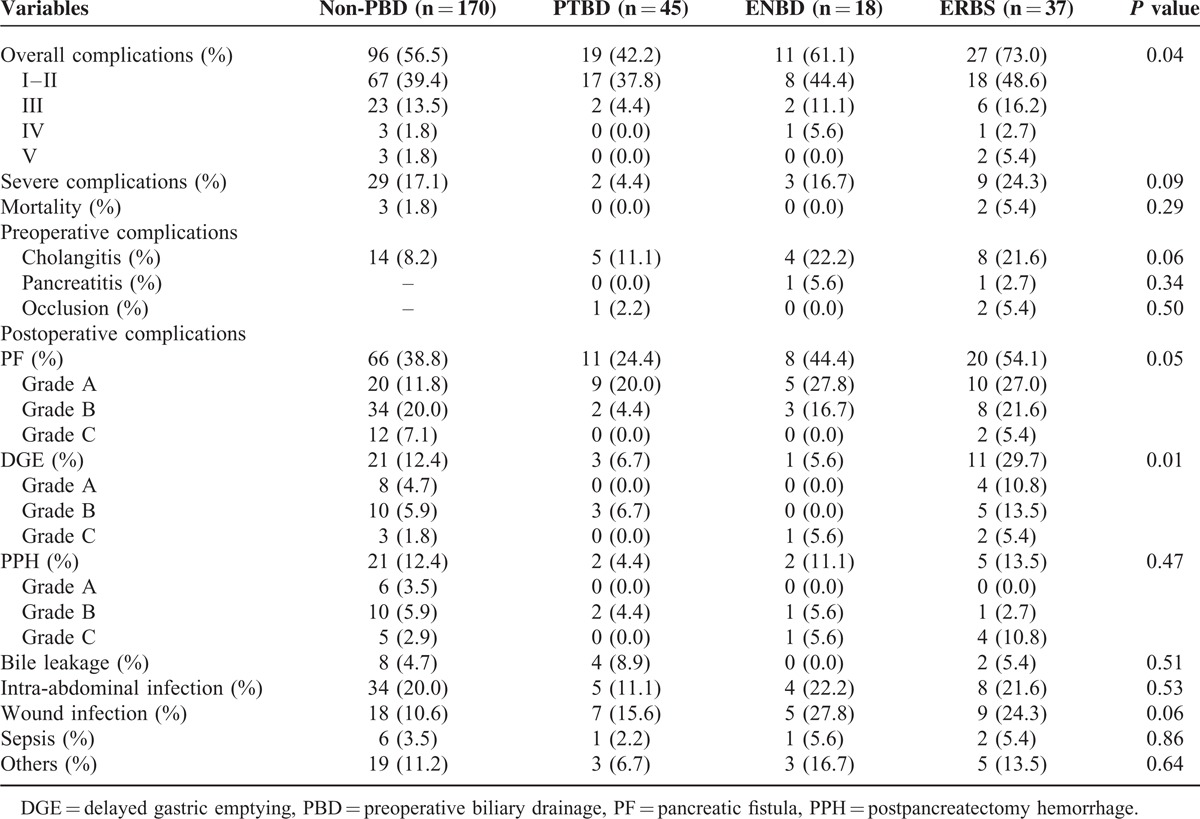
Comparisons of Perioperative Complications Among the 4 Groups

## DISCUSSION

The results from this study demonstrated that the effects of different PBD methods on perioperative complications differed widely in extrahepatic bile duct cancer patients who underwent pancreaticoduodenectomy. Although any drainage method can effectively relieve bile duct pressure and decrease serum ALT and TB levels, the high incidence of preoperative cholangitis and long period of PBD duration were disadvantages of the ERBS method. The incidence of severe complications following pancreaticoduodenectomy markedly decreased in patients receiving PTBD treatment, but the procedure of ERBS increased the rates of DGE and wound infection and prolonged postoperative hospital stay significantly. Moreover, there was no significant beneficial or adverse effect with regard to ENBD method. Therefore, PTBD may be the optimal PBD method for extrahepatic bile duct cancer patients with curative intent and ERBS is not advised.

PBD is introduced to reverse cholestatic liver status and improve postoperative outcome in patients with curative intent. All PBD methods, including PTBD, ENBD, and ERBS, can improve hepatic function after a period of drainage. However, the effect of PBD on patient outcome is controversial. It may be related to the benefits and drawbacks of each method. Internal drainage methods, such as ERBS, normalize bile flow in digestive tract which is important for improving metabolic and immune function and preventing bacterial translocation.^17,18^ Due to the direct connection between biliary tree and duodenum, micrograms and food debris can freely ascend into the biliary tree through the stent, resulting in stent occlusion and cholangitis.^19^ External drainage methods, including PTBD and ENBD, decompress biliary obstruction by draining bile flow outside the body, avoiding regurgitation of the intestinal contents. The common drawbacks of external drainage methods are the risk of tube dislodgement and the loss of body fluid which may affect the recovery of hepatic function and immunity. As an invasive technique, PTBD has the possibility of intrahepatic hemorrhage and infection. The procedure of ENBD, accomplished by endoscopic retrograde cholangiopancreatography, is also associated with drainage-related cholangitis.^20^

Based on the abovementioned analyses, it appears that ERBS is superior to PTBD and ENBD in hepatic function and patient outcome. On the contrary, internal drainage methods have been proved to be linked with a high incidence of perioperative complications in the clinical setting. Kitahata et al classified PBD methods as internal drainage group (ERBS) and external drainage group (PTBD and ENBD). The preoperative cholangitis rate in the internal drainage group was significantly higher than in the external drainage group.^21^ There were also investigations comparing PTBD with endoscopic biliary drainage methods which found that PTBD could rapidly decompress biliary obstruction with lower frequency of drainage-related complications.^22,23^ In the present study, the incidence of preoperative complications was similar between different PBD methods, but cholangitis occurred significantly higher in the ERBS group than the non-PBD group. The cholangitis rate in the PTBD group was the lowest among the drainage groups, but without any significant difference.

The incidence of postoperative complications following pancreaticoduodenectomy is related to a series of correlative risk factors. A study with multivariate analyses reported that development of preoperative cholangitis after PBD and small pancreatic duct were independent risk factors for postpancreatectomy PF.^24^ The occurrence of DGE after pancreaticoduodenectomy was associated with postoperative complications and PF.^25^ Also, there were reports that preoperative cholangitis could cause increased incidence of infectious complications.^26,27^ These conclusions show that preoperative cholangitis plays an important role in postoperative complications. It is not wondering that DGE and wound infection occurred more often in patients receiving ERBS treatment and postoperative hospital stay was prolonged. Therefore, biliary drainage with ERBS is not recommended for jaundiced patients with curative intent. Because the rates of preoperative cholangitis and overall complications in the PTBD group and non-PBD group were similar, the lower incidence of severe complications in the PTBD group demonstrated that biliary drainage with PTBD is an effective and safe procedure in decompressing biliary obstruction and improving patient outcome. Lower incidence of severe complications in the PTBD group could make patients recover faster than those of other groups and shorten postoperative hospital stay.

Besides the preoperative cholangitis, drainage duration can also impact on patient outcome by the development of preoperative complications. In general, a minimum of 4 to 6 weeks of PBD was advised. Long-term PBD could cause an extensive inflammatory reaction in the bile duct wall with increasing possibility of bacterial colonization of the biliary tree.^28^ Son et al reported that PBD duration <2 weeks, which was associated with lower rate of preoperative drainage-related complications, was more appropriate in severely jaundiced patients with periampullary cancer.^29^ However, the optimal duration from that study may not be appropriate because it did not distinguish different PBD methods. Compared with the ERBS group, PBD duration was significantly shorter in the PTBD and ENBD groups. Higher rates of preoperative complications may contribute to longer drainage duration in the ERBS group than other groups. No significant differences in perioperative complications were observed between the ENBD group and other groups. These results on ENBD are not convinced due to the small number of patients in the ENBD group.

Several factors may influence these results, including operative procedure, tumor location, and stents selection. Diener et al have concluded that there were no differences between PD and PPPD in postoperative mortality, morbidity and survival.^30^ In addition, no significant difference was observed in operative procedure of the 4 groups. Extrahepatic bile duct carcinoma was classified into middle and distal tumor in this study and the latter type can lead to the dilation of pancreatic duct or chronic pancreatitis which may decrease the rates of certain complications after ERCP and surgery. Tumor location showed no significant difference among the 4 groups. The selection of drainage stents is another issue in discussion. Some studies revealed that self-expandable metal stents provide better drainage compared with plastic stents.^31,32^ Researches have also reported that self-expandable metal stents were associated with more wound infections and longer operative time than plastic stents after surgery.^33^ Considering the cost effectiveness of various stents, plastic stents were used for all patients of the ERBS group in our center.

Recently, Lai et al concluded 3 questions about PBD: the necessity of PBD for jaundiced patients with curative intent, the optimal interval of PBD, and the most appropriate drainage method.^34^ Of the 3 questions, which method is the most appropriate one is considered to be the core issue. Through individual analyses of different PBD methods, our study answered 2 of them: biliary drainage with PTBD is necessary for extrahepatic bile duct cancer patients following pancreaticoduodenectomy and is the most appropriate drainage method.

There are several limitations to this study. First, due to the retrospective nature, the conclusions of this study may not be fully convinced. Second, the small number of patients in the ENBD group would influence the results of ENBD method. The lack of effects of different PBD methods on long-term postoperative outcomes is also shortcoming of this study. Furthermore, a prospective random trial should be designed and performed for valid and scientific conclusions of the effects of different PBD methods on complications.

In conclusion, with the advantages in reducing the incidence of severe complications without increasing the rates of preoperative cholangitis and overall complications, PTBD is an effective and safe method for extrahepatic bile duct cancer patients following pancreaticoduodenectomy and is best recommended. The ERBS method which is associated with high preoperative cholangitis and postoperative complication rates should be avoided. The efficiency of ENBD needs further analysis.
